# Adding Streptomycin to an Intensified Regimen for Tuberculous Meningitis Improves Survival in HIV-Infected Patients

**DOI:** 10.1155/2015/535134

**Published:** 2015-08-04

**Authors:** Gerardo Alvarez-Uria, Raghavakalyan Pakam, Manoranjan Midde, Pradeep Sukumar Yalla, Praveen Kumar Naik

**Affiliations:** Department of Infectious Diseases, Bathalapalli Rural Development Trust Hospital, Kadiri Road, Bathalapalli, Andhra Pradesh 515661, India

## Abstract

In low- and middle-income countries, the mortality of HIV-associated tuberculous meningitis (TM) continues to be unacceptably high. In this observational study of 228 HIV-infected patients with TM, we compared the mortality during the first nine months of patients treated with standard antituberculosis therapy (sATT), intensified ATT (iATT), and iATT with streptomycin (iATT + STM). The iATT included levofloxacin, ethionamide, pyrazinamide, and double dosing of rifampicin and isoniazid and was given only during the hospital admission (median 7 days, interquartile range 6–9). No mortality differences were seen in patients receiving the sATT and the iATT. However, patients receiving the iATT + STM had significant lower mortality than those in the sATT group (hazard ratio [HR] 0.47, 95% confidence interval [CI] 0.24 to 0.93). After adjusting for other covariates, the mortality hazard of the iATT + STM versus the sATT remained statistically significant (adjusted HR 0.2, 95% CI 0.09 to 0.46). Other factors associated with mortality were previous ATT and low albumin concentrations. The mortality risk increased exponentially only with CD4+ lymphocyte concentrations below 100 cells/*μ*L. In conclusion, the use of iATT resulted in a clinically important reduction in mortality compared with the standard of care only if associated with STM. The results of this study deserve further research.

## 1. Introduction

Over half of the patients with HIV-associated tuberculous meningitis die soon after diagnosis, and many of the survivors suffer from chronic neurological sequelae [1]. In spite of the poor prognosis, there has not been any major breakthrough in the chemotherapy of tuberculous meningitis in the last decades [2]. However, in recent years, there has been a growing interest in finding new intensified regimens that could result in improved survival [3–5]. In a phase two randomized trial investigating the effect of moxifloxacin and intravenous rifampicin during the first two weeks of treatment of tuberculous meningitis, higher exposure to rifampicin was associated with a survival benefit compared with a standard antituberculosis therapy (sATT) [3, 6].

Streptomycin (STM) was the first drug to reduce mortality in the treatment of tuberculous meningitis in the nineteen forties [7]. Interesting, the addition of isoniazid and* para*-aminosalicylic acid to STM in the nineteen fifties achieved similar survival rates to the ones achieved with the currently recommended ATT [8]. In more recent studies, STM resistance is associated with slower CSF clearance of mycobacteria and might be associated with poorer prognosis in patients with isoniazid resistance [9, 10]. However, the availability of less toxic drugs with better CSF penetration has limited the use of STM in the treatment of tuberculous meningitis [11].

In a previous study from our cohort, we observed a mortality reduction in HIV-associated tuberculous meningitis after implementation of an intensified ATT (iATT) [5]. However, some of the patients included in the iATT group in the previous study received also STM following the National Guidelines for the treatment of tuberculosis for patients with previous history of ATT (category 2 ATT) [12]. In this study, we aimed to assess the effect of STM on the effectivity of the iATT comparing three treatment groups: sATT, iATT, and iATT with STM (iATT + STM).

## 2. Methods

### 2.1. Setting and Design

The Vicente Ferrer HIV Cohort Study (VFHCS) is an open cohort study of HIV-infected patients who have attended the Rural Development Trust Hospital in Bathalapalli, Anantapur District, AP, India. The hospital provides medical care free of charge to people living with HIV. In our setting, 72% of the population live in rural areas [13]. The HIV epidemic is largely driven by heterosexual transmission and it is characterized by low CD4 cell counts at presentation, poor socioeconomic conditions, and high levels of illiteracy [14–16].

For this study, we included all HIV-infected patients diagnosed with tuberculous meningitis from 1 January 2011 to 1 October 2014 from the VFHCS database. The selection of patients from the database was executed on 14 March 2015. Patients who did not meet the proposed criteria for definite, probable, or possible tuberculous meningitis were excluded from the analysis [17].

### 2.2. Treatment

The management of tuberculous meningitis in our cohort has been described in detail elsewhere [5]. All patients with tuberculous meningitis were admitted to the hospital. Before 29 January 2012, patients were treated with a sATT (isoniazid 300 mg, rifampicin 450 mg, ethambutol 800 mg, and pyrazinamide 1500 mg) and, after 29 January 2012, patients received an iATT (isoniazid 600 mg, rifampicin 900 mg, pyrazinamide 1500 mg, levofloxacin 750 mg, and ethionamide 750 mg) while admitted (sATT was given after discharge). Following Indian Guidelines for tuberculosis, in patients who had received ATT for at least one month in the past, 750 mg intramuscular streptomycin was added during the first two months of ATT [12]. Intravenous dexamethasone was given and was rapidly tapered during admission. Patients not on ART at the time of ATT initiation were counselled to start ART after 14 days of hospital discharge.

### 2.3. Statistical Analysis

We used time-to-event methods to study the mortality during the first nine months after the diagnosis of tuberculous meningitis. Time was measured from ATT initiation to death. Patients who did not die during the study period were censored at nine months or at their last visit date, whichever occurred first. Univariate and multivariate analyses were performed using Cox proportional hazard models. The proportional hazard assumption was assessed performing log-log survival curves based on Schoenfeld residuals [18]. The log-linearity assumption was checked for all continuous variables. Continuous variables that did not have a linear relationship with the log-hazard were transformed using restricted cubic splines with four knots [19]. As the coefficients for restricted cubic splines are difficult to interpret, the relationship of continuous covariates with the event of interest was presented graphically [20]. Statistical analysis was performed using Stata Statistical Software (Stata Corporation; Release 12.1. College Station, Texas, USA). The VFHCS was performed according to the principles of the Declaration of Helsinki and was approved by the Ethics Committee of the Rural Development Trust Hospital.

## 3. Results

During the study period, 228 patients were diagnosed with tuberculous meningitis; 79 received the sATT, 119 received the iATT, and 30 received iATT + STM. The median duration of the iATT was 7 days (interquartile range [IQR], 6–9). Weight was measured in 63 patients in the sATT group, 88 patients in the iATT group, and 27 patients in the iATT + STM group, and the median weight was 46 kg (IQR, 42–53), 49 kg (IQR, 41–55), and 46 kg (IQR, 41–53), respectively. Thus, the median dose of rifampicin was 9.8 mg/kg (IQR, 8.5–10.7) in the sATT group, 18.4 mg/kg (IQR, 16.4–22) in the iATT group, and 19.6 mg/kg (IQR, 17–22) in the iATT + STM.

Baseline characteristics of patients by treatment group are presented in Table 1. The proportion of patients on antiretroviral therapy (ART) at the time of ATT initiation was higher in the iATT + STM group (56.3%) than in the sATT group (32.9%) and in the iATT group (25.2%). Other differences were not statistically significant. Eighteen patients (20.8%) in the sATT group had been treated previously for tuberculosis.

**Figure 1:**
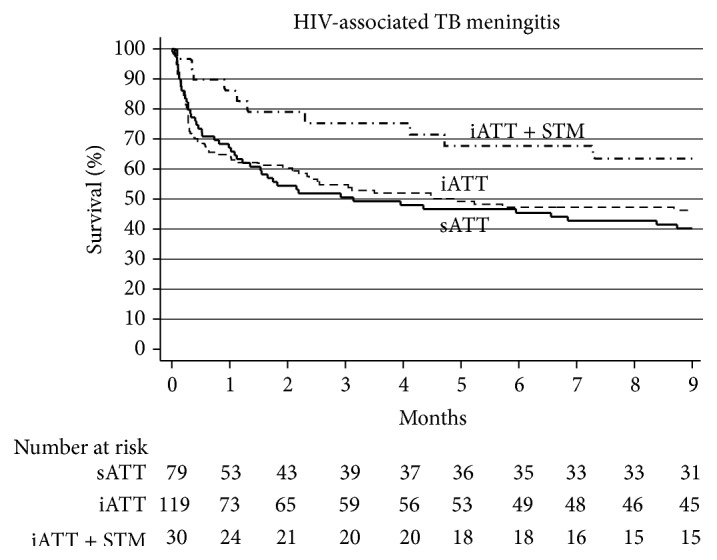
Kaplan-Meier survival estimates by treatment group. iATT, intensified antituberculosis therapy; sATT, standard antituberculosis therapy; STM, streptomycin.

**Figure 2:**
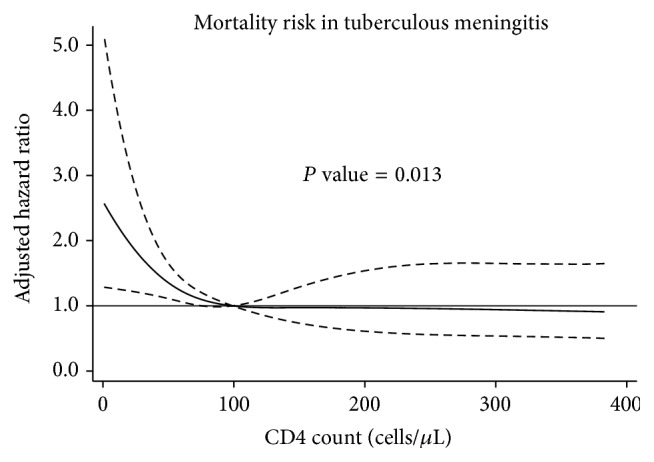
Adjusted hazard ratio and 95% confidence interval for mortality according to CD4+ lymphocytes using restricted cubic splines.

Kaplan-Meier survival estimates by treatment group are shown in Figure 1, and univariate and multivariate analysis of factors associated with mortality are presented in Table 2. In the univariate analysis, patients in the sATT and iATT groups had similar mortality risk (iATT versus sATT hazard ratio [HR] 0.90, 95% confidence interval [CI] 0.62 to 1.32). Patients in the iATT + STM had lower mortality risk than patients in the sATT group (HR 0.47, 95% CI 0.24 to 0.93), and the mortality difference at nine months was 23.3% (95% CI 2.1 to 44.5). In the multivariate analysis, being previously treated for tuberculosis (adjusted HR [aHR] 3.23, 95% CI 1.68 to 6.24), low albumin concentrations (aHR 0.74 per increase of 1 g/dL, 95% CI 0.58 to 0.95), and low CD4+ lymphocyte concentrations (Figure 2) were independently associated with increased mortality. The risk of death increased exponentially only with CD4+ lymphocyte concentrations below 100 cells/*μ*L (*P* value = 0.013). Compared with the sATT, the use of the iATT + STM was associated with a reduced risk of mortality (aHR 0.2, 95% CI 0.09 to 0.46), but the use of iATT without STM was not (aHR 1.14, 95% CI 0.74 to 1.76). In a sensitivity analysis, we removed being previously treated for tuberculosis from the multivariate model, and the use of iATT + STM remained significantly associated with reduced mortality (aHR 0.5, 95% CI 0.25 to 0.99).

## 4. Discussion 

These results complement the findings from a previous study of our cohort where we observed a mortality reduction in HIV-associated tuberculous meningitis after implementation of the iATT [5]. After adjusting for STM use, the iATT was only effective in reducing mortality when combined with STM.

The results of previous studies suggest that rifampicin is the most important drug among those included in the iATT [3, 6]. Unlike in a previous clinical trial using intravenous rifampicin for 14 days [3], we did not observe a survival benefit when the iATT was given without STM. In a recent clinical trial comparing the bactericidal effect of the standard dose of rifampicin (10 mg/kg) with higher doses, an improved bactericidal activity was achieved only with rifampicin dosing equal to or greater than 30 mg/kg [21]. It is possible that the oral dose of rifampicin used in our study (near 20 mg/kg) was not high enough to result in a higher bactericidal activity.

STM was given only to patients previously treated for tuberculosis, which is a known risk factor for mortality and poor outcomes [16, 22, 23]. Given the poor CSF penetration of STM [24, 25], the beneficial effect on survival of STM and the iATT is intriguing. In combination with a standard dose of rifampicin (10 mg/kg) in patients with pulmonary tuberculosis, streptomycin has a strong bactericidal activity during the first six days of ATT [26]. Our results suggest that STM and higher doses of rifampicin could have a synergetic effect against tuberculosis, but new studies are needed to confirm this hypothesis.

The use of restricted cubic splines allowed for a flexible representation of the relationship between the concentrations of CD4+ lymphocytes and mortality. The mortality hazard increased exponentially with lower CD4+ lymphocyte concentration, but only in patients with CD4+ lymphocytes <100 cells/*μ*L. This nonlinear association between CD4+ lymphocytes and the log-hazard should be taken into account in future clinical trials or studies investigating prognostic factors of HIV-associated tuberculous meningitis [27].

The study has some limitations. Observational studies can be biased due to unknown confounders not included in the multivariate analysis. Unlike in randomized clinical trials, treatment groups were not uniformly balanced, and patients in the iATT + STM group were more likely to be on ART at the time of ATT initiation. In addition, we did not have information about the drug sensitivity of mycobacteria among treatment groups or the severity of the clinical condition of the patients. On the other hand, we did not exclude patients because of the severity of tuberculous meningitis, so the study reflects the “real-life” of HIV-associated tuberculous meningitis in a resource-limited setting.

## 5. Conclusions

In this cohort study in a resource-poor setting, the use of iATT resulted in reduced mortality only when combined with STM in HIV-infected patients with tuberculous meningitis. Because the study is observational in nature, we should be cautious about our findings. However, the mortality reduction was clinically important, and the results of this study deserve further research.

## Figures and Tables

**Table 1:** Baseline characteristics by treatment group.

	Standard ATT	Intensified ATT	Intensified ATT + STM	*P* value
Gender				0.19
Male	52 (65.82)	86 (72.27)	25 (83.33)	
Female	27 (34.18)	33 (27.73)	5 (16.67)	
On ART				0.004
No	53 (67.09)	89 (74.79)	13 (43.33)	
Yes	26 (32.91)	30 (25.21)	17 (56.67)	
Age (years), median (IQR)	36.3 (31.6–45.3)	37.1 (30.9–50)	33.2 (30–40.4)	0.33
Albumin (g/dL), median (IQR)	3.5 (2.7–4)	3.4 (2.9–3.9)	3.7 (3.1–4.4)	0.21
CD4 count (cells/*μ*L), median (IQR)	86 (45–174)	102 (47–173)	103 (58–169)	0.65

ART, antiretroviral therapy; ATT, antituberculosis therapy; IQR, interquartile range; STM, streptomycin.

Data are presented as number (%) unless otherwise indicated. *P* values were calculated using chi^2^ test for categorical variables and Kruskal-Wallis rank test for continuous variables.

**Table 2:** Univariate and multivariate analyses of mortality using Cox proportional hazard models.

	HR (95% CI)	aHR (95% CI)
Intensified versus standard ATT	0.90 (0.62–1.32)	1.14 (0.74–1.76)
Intensified + STM versus standard ATT	0.47^*∗*^ (0.24–0.93)	0.20^*∗*^ (0.09–0.46)
Female	0.87 (0.58–1.31)	0.79 (0.52–1.20)
On ART	0.60^*∗*^ (0.39–0.91)	0.64 (0.40–1.02)
Previous ATT	1.02 (0.66–1.59)	3.23^*∗*^ (1.68–6.24)
Age (years)	1.00 (0.98–1.02)	1.00 (0.98–1.01)
Albumin (g/dL)	0.66^*∗*^ (0.53–0.83)	0.74^*∗*^ (0.58–0.95)

^*∗*^ *P* value <0.05; aHR, adjusted hazard ratio; ART, antiretroviral therapy; ATT, antituberculosis therapy; HR, hazard ratio; STM, streptomycin. Adjusted hazard ratios are also adjusted for CD4 cell counts using restricted cubic splines (see Figure 2).
